# Interactive exploration of a global clinical network from a large breast cancer cohort

**DOI:** 10.1038/s41746-022-00647-0

**Published:** 2022-08-10

**Authors:** Nadir Sella, Anne-Sophie Hamy, Vincent Cabeli, Lauren Darrigues, Marick Laé, Fabien Reyal, Hervé Isambert

**Affiliations:** 1 Institut Roche, Boulogne-Billancourt, France; 2 Residual Tumor & Response to Treatment Laboratory, RT2Lab, INSERM, U932 Immunity and Cancer, Universite Paris CiteInstitut Curie, Paris, 75248 France; 3 Laboratoire Physico Chimie Curie, Institut Curie, PSL Research University, CNRS UMR168, Paris, 75005 France; 4 Department of Medical Oncology, Universite Paris Cite, Institut Curie, Saint-Cloud, 92230 France; 5 Department of Surgery, Institut Curie, Université Paris Cité, Paris, 75248 France; 6 Department of Tumor Biology, Institut Curie, Paris, 75248 France; 7 Department of Pathology, Henri Becquerel Cancer Center, INSERM U1245, UniRouen Normandy University, Rouen, France

**Keywords:** Outcomes research, Risk factors, Breast cancer

## Abstract

Despite unprecedented amount of information now available in medical records, health data remain underexploited due to their heterogeneity and complexity. Simple charts and hypothesis-driven statistics can no longer apprehend the content of information-rich clinical data. There is, therefore, a clear need for powerful interactive visualization tools enabling medical practitioners to perceive the patterns and insights gained by state-of-the-art machine learning algorithms. Here, we report an interactive graphical interface for use as the front end of a machine learning causal inference server (MIIC), to facilitate the visualization and comprehension by clinicians of relationships between clinically relevant variables. The widespread use of such tools, facilitating the interactive exploration of datasets, is crucial both for data visualization and for the generation of research hypotheses. We demonstrate the utility of the MIIC interactive interface, by exploring the clinical network of a large cohort of breast cancer patients treated with neoadjuvant chemotherapy (NAC). This example highlights, in particular, the direct and indirect links between post-NAC clinical responses and patient survival. The MIIC interactive graphical interface has the potential to help clinicians identify actionable nodes and edges in clinical networks, thereby ultimately improving the patient care pathway.

## Introduction

The availability of health data from patient medical records is increasing, and these data constitute, in theory, a rich resource for research purposes. However, despite the unprecedented amount of information now available, health data remain underexploited due to their heterogeneity and complexity. There is, therefore, an urgent need for innovative tools, based on intuitive and interactive graphical interfaces, specifically designed for the exploration of health data by medical practitioners. Data visualization is gradually emerging as a new field of research, and graphical representations are used for two main purposes: (i) explanatory illustration, to highlight novel scientific insights graphically and to ensure efficient communication between scientists^1–4^; and (ii) exploratory analysis, searching for relationships previously overlooked and leading to new discoveries, thereby maximizing the potential of information-rich databases. We present here an *exploratory analysis* of a global clinical network from a large breast cancer cohort, with a novel interactive graphical interface for the exploration of health data.

We previously developed an advanced computational method for graphical analyses, including causal relationships, from multivariate data^5^. The underlying MIIC (Multivariate Information-based Inductive Causation) algorithm, which was released as an online server^6^, uses a machine learning method combining constraint-based and information theory approaches to reconstruct causal, non-causal or mixed networks from large datasets. The MIIC algorithm was first developed to analyze categorical genomic data^5,6^ and has recently been extended to the analysis of more challenging heterogeneous datasets, such as medical records, combining both categorical and continuous variables, in which interdependence is notoriously difficult to assess^7^.

Breast cancer (BC) clinical datasets are particularly suitable for the type of exploratory analysis presented here, as BC is a complex heterogeneous disease highly variable in its aggressiveness and prognosis. BC remains one of the leading causes of cancer-related death among women. The BC patients included in the cohort analyzed here were treated with neoadjuvant (or preoperative) chemotherapy (NAC). NAC was originally restricted to patients with inflammatory or locally advanced BC, but is now the standard care for aggressive early-stage breast cancers, i.e., triple-negative (TNBC) and *HER2*-positive BCs^8,9^. From the patient’s viewpoint, the benefits of the neoadjuvant strategy include a greater feasibility of breast-conserving surgery and the prognostic stratification of risk obtained after analyses of the residual tumor burden at surgery. From the research and development standpoint, the neoadjuvant setting makes it possible to monitor the chemosensitivity of the tumor in vivo, and provides an opportunity for the rapid validation of research hypotheses and the acceleration of drug approval.

## Results

**Fig. 1:**
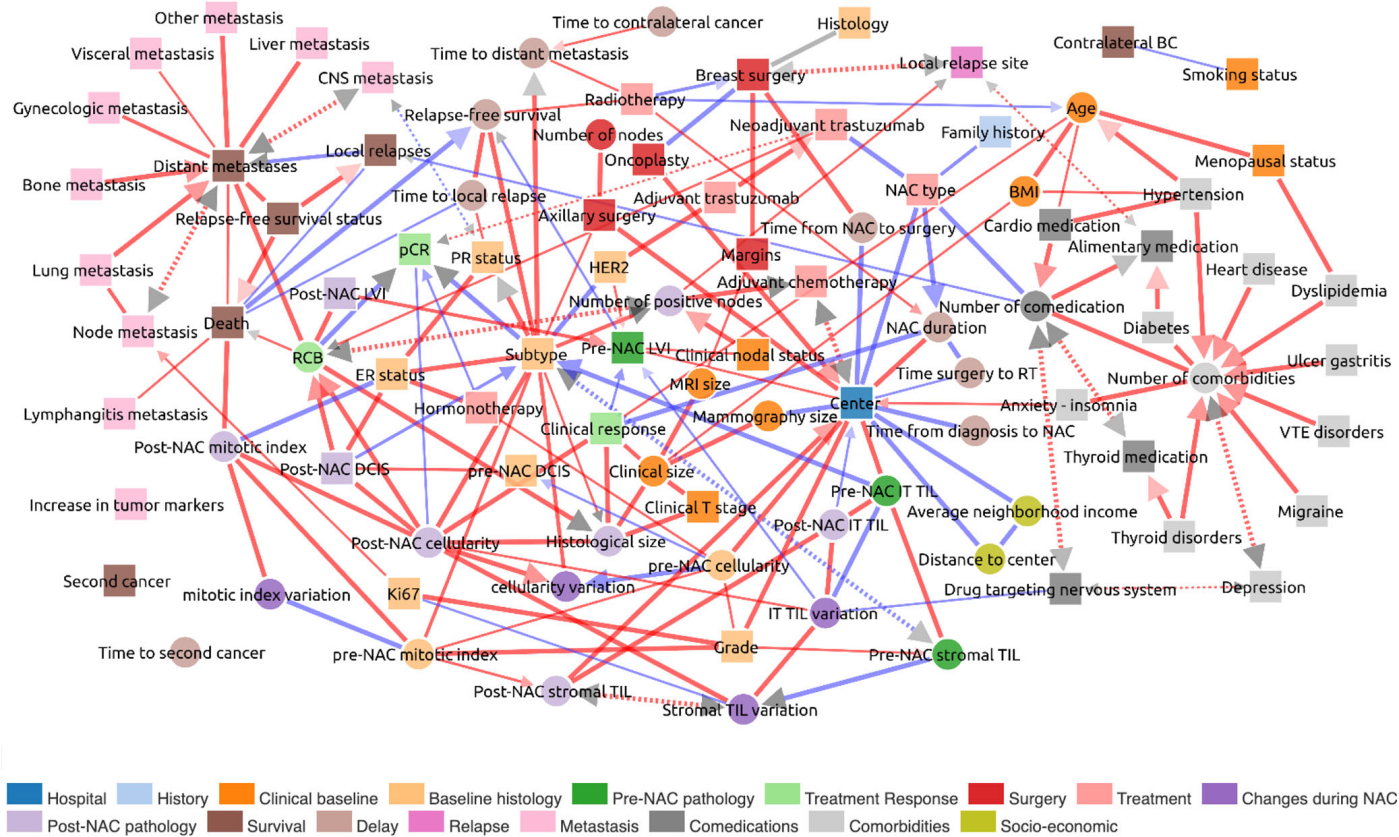
MIIC global network for the NEOREP breast cancer cohort. Each node corresponds to a variable of the dataset, with circles indicating continuous variables and squares indicating categorical variables. The colors define a category of variables, as detailed under the figure. Each edge corresponds to a “direct” association between two variables with different types of orientation described in Methods. BC breast cancer, BMI body mass index, DCIS ductal carcinoma in situ, ER estrogen receptor status, LVI lymphovascular invasion, NAC neoadjuvant chemotherapy, CNS central nervous system, pCR pathological complete response, PR progesterone receptor status, RCB residual cancer burden, TILs tumor-infiltrating lymphocytes. Blue edges indicate negative partial correlations, red edges indicate positive partial correlations.

The global network displayed in Fig. 1 is accessible at https://miic.curie.fr/job_results_showcase.php?id=NEOREP. We discuss below some of the links inferred in the NEOREP network after grouping according to several clinically relevant concepts identified from published studies on BC.

### MIIC performs quality control

MIIC first identifies relationships between a disease and the corresponding treatment. ER positivity—which is predictive of efficacy for anti-hormonal treatment^10^—is associated with the use of endocrine therapy (Supplementary Fig. 3A), and a similar association is observed for *HER2*-positivity and trastuzumab use (Supplementary Fig. 3B)^11^. Beyond cancer, significant associations are also found between depression and the use of psycholeptics (Supplementary Fig. 3C), between thyroid disorders and thyroid hormone use (Supplementary Fig. 3D), and between hypertension and drugs for the treatment of cardiovascular diseases (Supplementary Fig. 3E). More generally, comedication use is associated with the type of NAC (Supplementary Fig. 3F), reflecting the greater likelihood of less toxic regimens being prescribed to fragile patients (patients on other types of medication) than to patients without comedication^12–14^.

MIIC then identifies clinical factors known to be epidemiologically related (Supplementary Fig. 4A). Menopause, a process occurring in older women, is directly linked to age (Supplementary Fig. 4B) (median age: 43 years for premenopausal, versus 58 years for postmenopausal women). Postmenopausal status is associated with dyslipidemia (Supplementary Fig. 4C)^15^. Consistent with these associations, body mass index (BMI) increases with age (Supplementary Fig. 4A, D) and both factors, which have been reported to increase cardiovascular risks, are linked to hypertension (Supplementary Fig. 4A, E). The number of drugs taken by a patient (comedication) increases with the number of comorbidities (Supplementary Fig. 4A, F).

### MIIC identifies inherent associations between variables

**Fig. 2:**
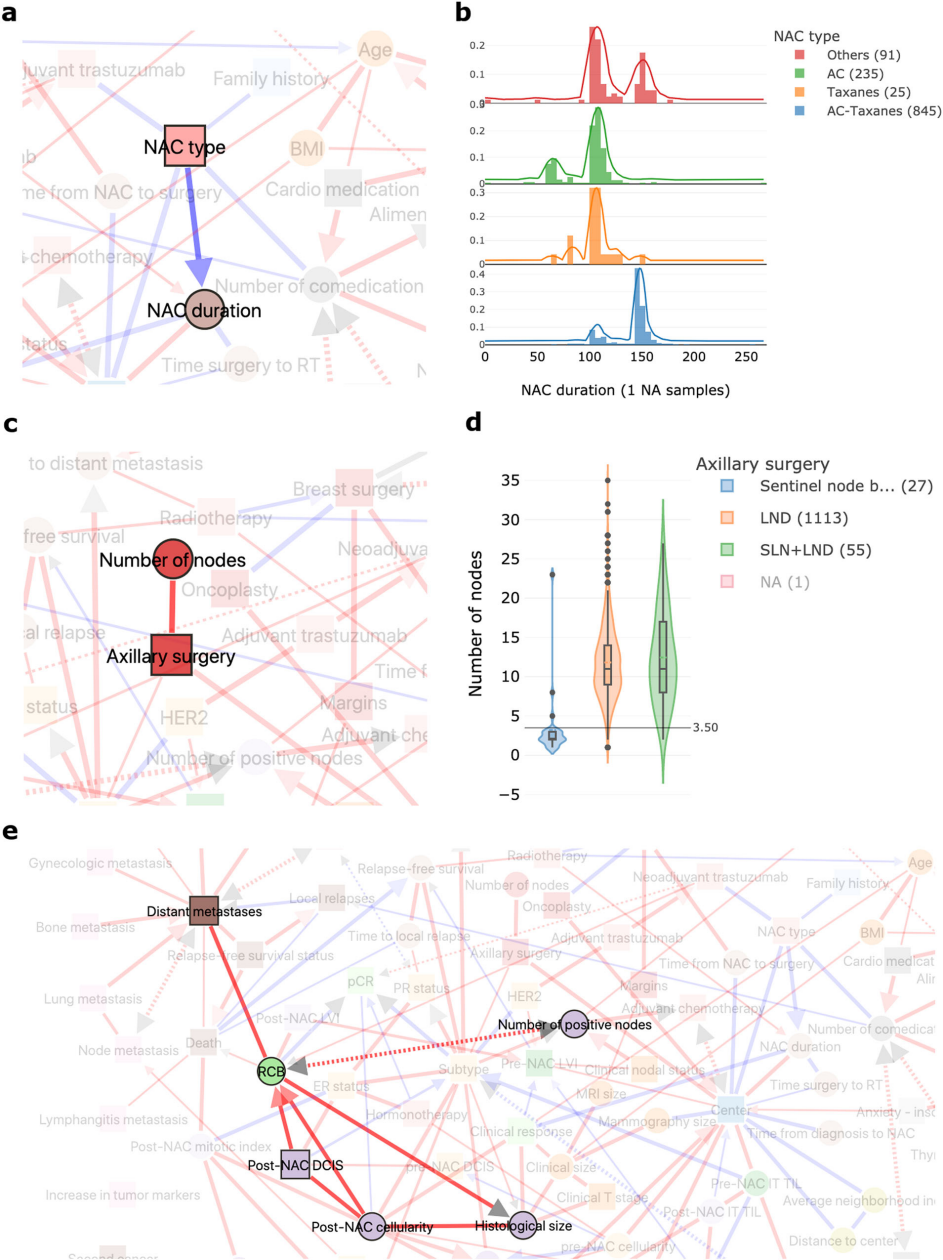
The MIIC interactive online interface identifies inherent associations between variables. **a** NAC type is directly correlated with NAC duration. NAC = neoadjuvant chemotherapy. **b** Distribution of neoadjuvant chemotherapy (NAC) duration (in days) according to the NAC regimen administered: anthracyclines (AC), taxanes or sequential AC-taxanes. **c** The number of axillary nodes in the histological specimen depends on the type of axillary surgery performed. **d** Boxplot showing the number of axillary nodes removed according to the type of surgery performed: lymph node dissection (LND), sentinel lymph node biopsy (SLN) or both. The boxes represent the IQR, the horizontal lines correspond to the median values and lines ends mark the upper and lower fence. The number of cases considered for the analysis are reported in the legend. **e** Network interactions of the RCB node with the five patterns making up the RCB score.

The duration of neoadjuvant treatment is directly linked to the type of NAC regimen delivered (Fig. 2a) reflecting the fact that anthracycline-based (AC) regimens usually include four cycles (median of 106 days, Fig. 2b), whereas sequential regimens in which anthracyclines are followed by taxanes are generally administered over six or eight cycles (median of 147 days, Fig. 2b). The number of nodes retrieved is associated with the type of axillary surgery (Fig. 2c), consistent with the fact that sentinel node (SLN) biopsy procedures were developed to reduce the number of lymph nodes removed during dissection (LND) (Fig. 2d)^16^. MIIC correctly represents the direct links between residual cancer burden (RCB) (Fig. 2e) and the patterns making up this score, derived from measurements on the primary tumor bed (size, fraction of invasive cancer, cellularity) and the regional lymph nodes (number of positive lymph nodes).

### MIIC identifies intra- and inter-modality associations

For the variables derived from pathology records, MIIC found associations between tumor grade, Ki67, and mitotic index (Supplementary Fig. 5A–C), all of which are markers of tumor proliferation^17^. MIIC can also visualize links between patterns assessed in different ways. Measurements of pre-NAC tumor size evaluated clinically, by mammography and by MRI, were found to be closely related (Supplementary Fig. 5C–E) as previously reported^18,19^. Similarly, the response to treatment assessed clinically at NAC completion was found to be associated with histological size based on the surgical specimen (Supplementary Fig. 5F).

### MIIC provides insight into tumor biology and response to treatment

The presence of lymphovascular invasion (LVI) in the post-NAC specimen is associated with a higher RCB index, consistent with the strong resistance to chemotherapy of these tumors^20^ (Supplementary Fig. 6A). TNBCs and *HER2*-positive tumors have a higher pre-NAC mitotic index and more stromal TIL infiltration (Supplementary Fig. 6B, C) than luminal BCs^21,22^. Consistently, high TIL levels are significantly associated with histological grade 3 tumors (Supplementary Fig. 6D).

### MIIC reflects clinical practice

Several associations highlighted in the network reflect clinical practice decisions applied throughout BC centers. For example, the likelihood of performing conservative breast surgery depends on tumor histology (higher rates of mastectomy have been reported for patients with lobular or other histological types of tumor less likely to respond to NAC)^23,24^ (Supplementary Fig. 7A) and is positively associated with the practice of oncoplastic surgery^25^ (Supplementary Fig. 7B). Similarly, lumpectomy is more frequently associated with radiation therapy than with mastectomy (Supplementary Fig. 7C)^26–29^. After surgery, the addition of a second line of treatment by adjuvant chemotherapy, to decrease the risk of relapse, is driven by the identification of factors associated with a poor prognosis^30^, such as high levels of lymph node involvement (Supplementary Fig. 7D).

**Fig. 3:**
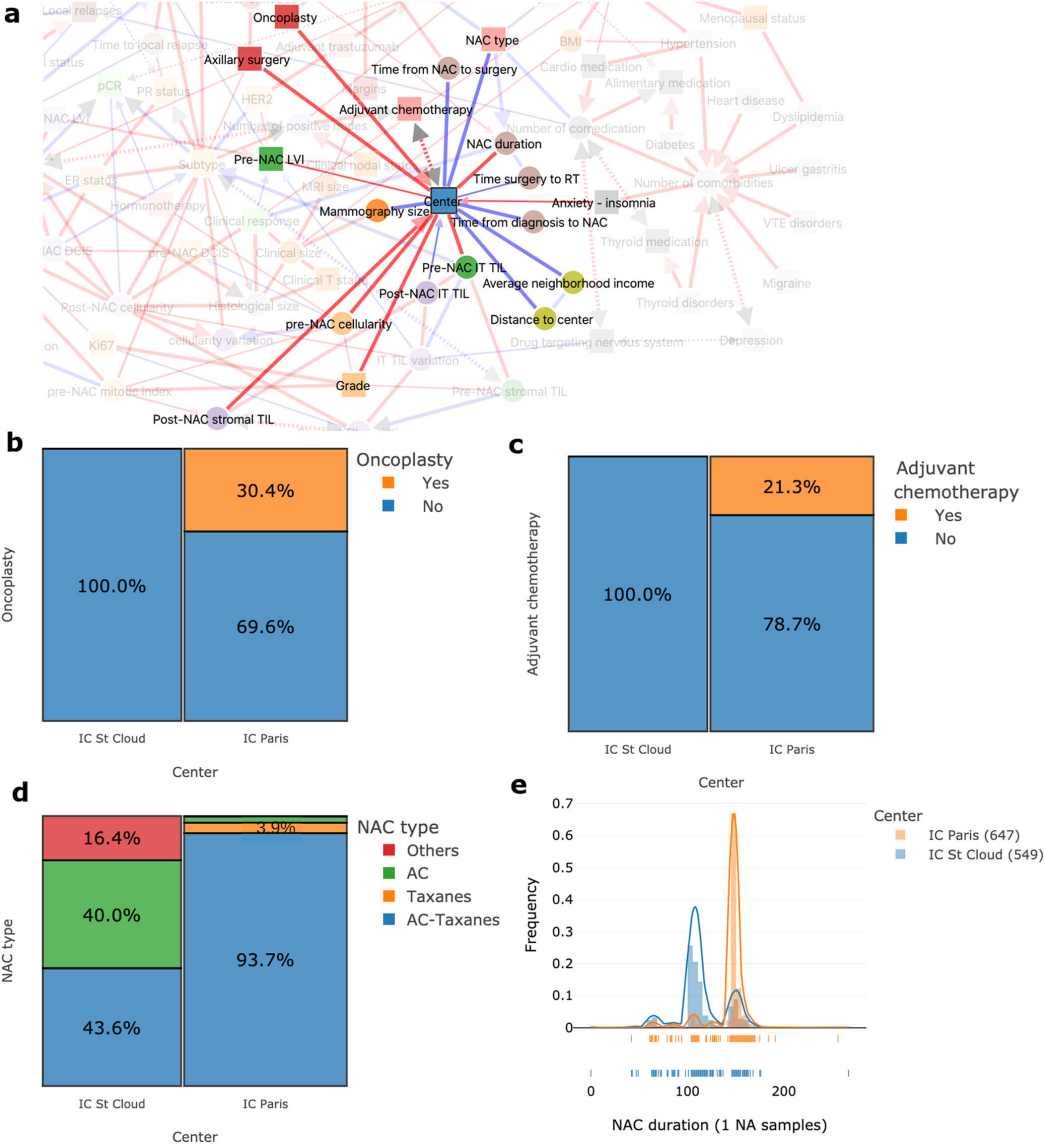
MIIC identifies differences in clinical practices between the two centers of the cohort. **a** Network interactions around the node “center” of treatment. **b** Proportion of patients undergoing oncoplastic surgery, according to treatment center: Paris or St Cloud. **c** Proportion of patients receiving adjuvant chemotherapy according to treatment center: Paris or St Cloud. **d** Proportion of the various NAC regimens according to treatment center. **e** Distribution plot for NAC duration in days, according to treatment center.

Beyond these well-established practices, MIIC also identified differences in clinical practices between the two centers of the cohort (Fig. 3a). For example, oncoplastic surgery and adjuvant chemotherapy were performed at only one of the two centers (Fig. 3b, c); the NAC regimen also differed between centers, with the Curie St Cloud center using more AC regimens than AC-taxane combinations, resulting in a shorter duration of NAC treatment (Fig. 3d, e).

### MIIC traces the natural course of the disease

**Fig. 4:**
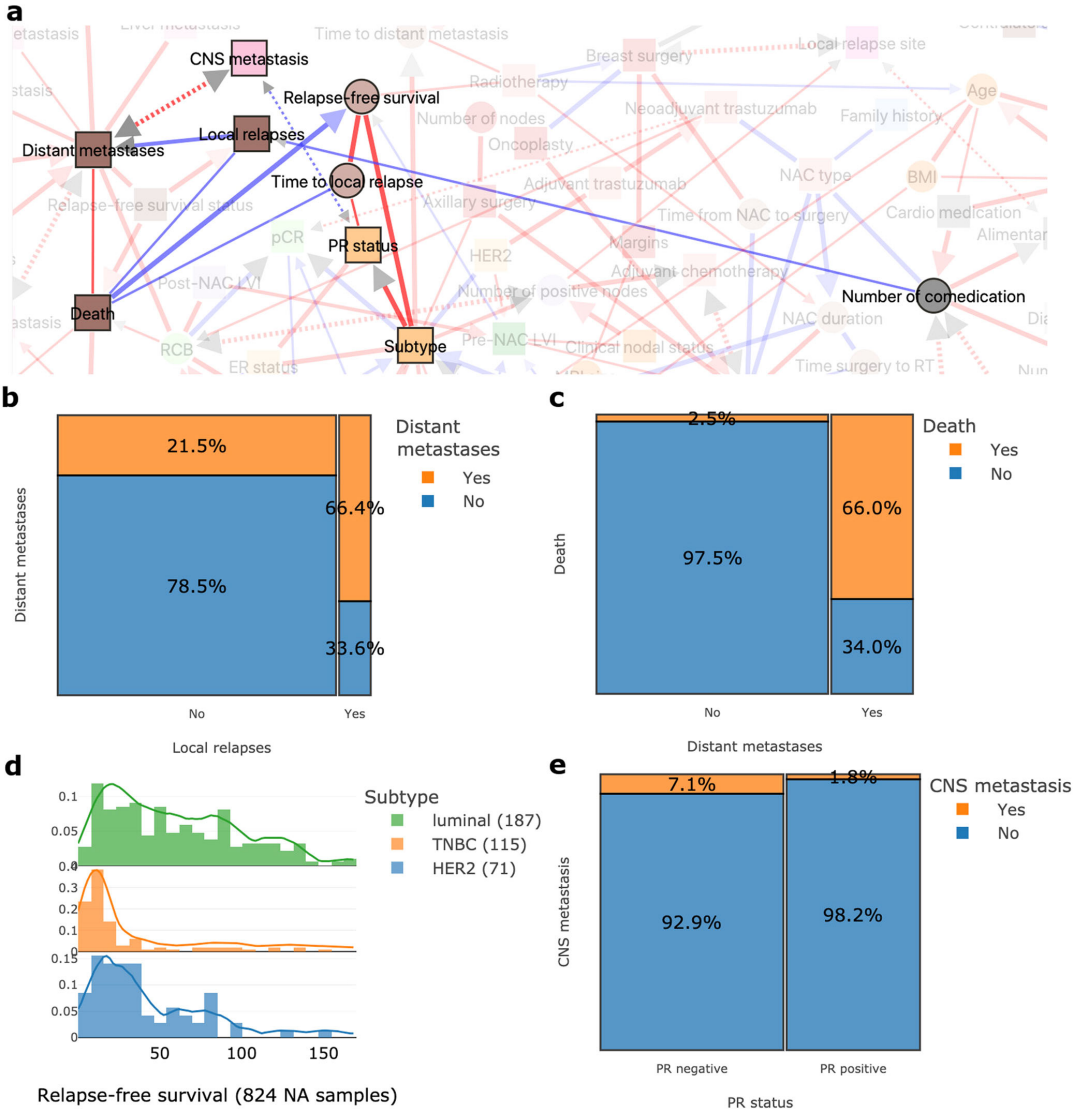
MIIC traces the natural course of the disease. **a** Network interactions showing links between relapses, metastases and death in breast cancer. **b** Proportion of distant metastases according to the occurrence or absence of local relapses. **c** Proportion of deaths according to distant metastasis status. **d** Distribution plot for relapse-free survival (in months) according to breast cancer subtype. **e** Proportion plot displaying the relationship between central nervous system (CNS) metastasis and progesterone receptor (PR) status.

The natural course of BC may include local relapse, possibly followed by distant metastases, the trigger events leading to death^31–35^ (Fig. 4a–c). Contralateral BC is often used in composite survival endpoints, such as distant relapse-free survival^36^, but MIIC clearly identifies contralateral BC as an event being independent of other oncologic events and almost totally isolated from the rest of the network (Fig. 1). Luminal BC is known to recur and develop metastases later than *HER2*-positive BC and TNBC (Fig. 4d)^21,22,37,38^. The link between has also been found between PR negativity and a higher risk of brain metastasis^39–43^ (Fig. 4e).

### MIIC identifies unexpected associations, leading to new discoveries

With more than 15 associations involving treatment center (Fig. 3a), MIIC unmasked an unexpected “batch” effect relating to the site of BC treatment in this cohort. The observed differences reflect not only differences in therapeutic practice, but also in the characteristics of the population (differences in the proportion of women with psychological disorders, difference in incomes), in tumor presentation (tumor size), in pathological variable scoring (grade, presence of pre-NAC LVI, tumor cellularity, TILs), and in time to treatment within the care pathway.

### MIIC identifies factors likely to improve prediction or prognosis

**Fig. 5:**
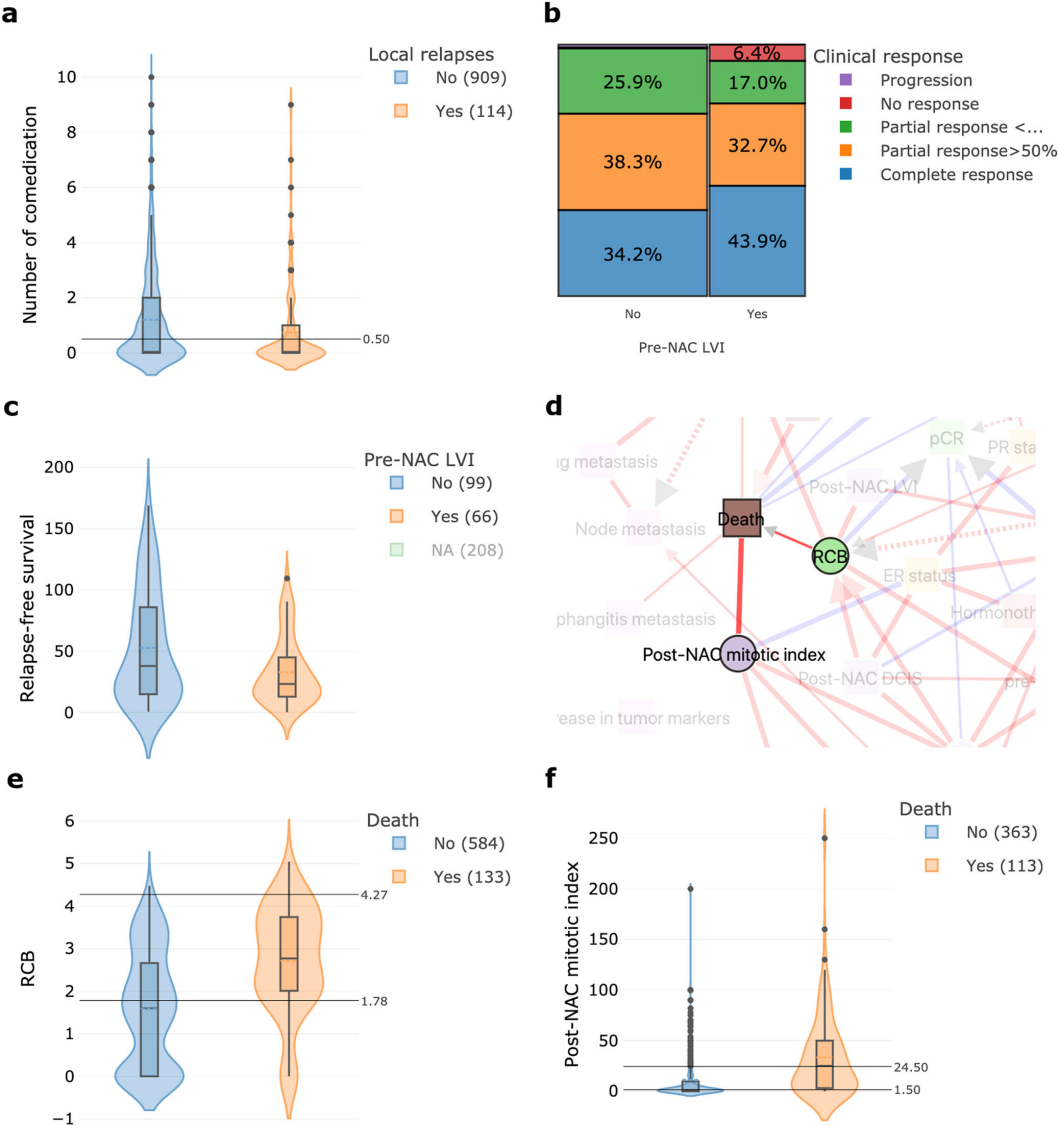
MIIC identifies factors likely to improve prediction or prognosis. **a** Network interaction displaying the link between local relapse occurrence and the number of drugs taken (comedication). **b** Proportion plot showing the percentage of different clinical responses according to the presence or absence of pre-NAC lymphovascular invasion. **c** Boxplot of relapse-free survival according to the presence or absence of pre-NAC lymphovascular invasion. **d** Network interaction displaying the link between death, RCB and post-NAC mitotic index. **e** Boxplot of RCB values according to vital status. **f** Boxplot of post-NAC mitotic index according to vital status. The boxes represent the IQR, the horizontal lines correspond to the median values and lines ends mark the upper and lower fence. The number of cases considered for the analysis are reported in the legend.

MIIC also favors new insights, e.g., comedication appears to protect against local relapse (Fig. 5a). Several retrospective studies have reported this association, with the use of statins^44^, NSAIDs^45^, or beta-blockers^46^ found to have indirect anticarcinogenic effects. It has recently been suggested that these non-oncological treatments may have immunomodulatory and chemosensitizing effects^47^.

### MIIC suggests relevant combinations of predictive of prognostic biomarkers

MIIC may provide clues to combinations of new prognostic biomarkers likely to improve the prediction of response to chemotherapy, or post-NAC prognosis. Pre-NAC lymphovascular invasion (LVI) was found to be associated with both lower rates of clinical response (Fig. 5b) and shorter relapse-free survival (Fig. 5c). Both RCB (Fig. 5d, e) and post-NAC mitotic index (Fig. 5d–f), a parameter rarely used in practice but nevertheless reported to be a predictor of BC recurrence^48,49^, appear to be strongly associated with the risk of death. MIIC may, therefore, be an efficient tool for identifying features likely to improve prognosis, by combining gold standard indicators with other parameters, such as post-NAC mitotic index, and post-NAC LVI, for example. Finally, MIIC also makes it possible to optimize the binning of residual cancer burden (RCB). RCB is a post-NAC histological score calculated as an increasing continuous index, and then subdivided into four classes (0, I, II, and III)^50^. Our analysis based on information maximization principles suggested a new unsupervised classification of RCB scores into three categories (Fig. 5e), with RCB = 0 with low RCB values merged, in particular, into a single class associated with a good prognosis.

## Discussion

When applied to a large cohort of BC patients, the MIIC algorithm successfully (i) performed quality controls; (ii) identified intra- and inter-modality correlations; (iii) highlighted differences in clinical practice, including center specificities; (iv) traced the natural course of the disease; (v) highlighted unsuspected and hidden associations, leading to new discoveries. The interactive visualization and causal analyses provided by this algorithm make it a promising tool for fast and effective explorations of the increasing amount of available health data.

The amount of exploitable health data is increasing exponentially. The best known health data resource for cancer studies remains the SEER (Surveillance, Epidemiology, and End Results) database, which collects data from population-based cancer registries covering approximately 34.6% of the US population^51,52^. By 2016, the National Cancer Database (NCDB) had amassed more than 34 million hospital records from cancer patients (almost four times the size of the SEER database), to become the largest clinical cancer registry in the world^53^. In France, the French administrative healthcare database, the SNDS (*Système National des Données de Santé*), is one of the largest administrative databases in the domain of medicine, providing many opportunities for medical research^54,55^, as it covers 99% of the French population (about 66 million people). The French government is planning to ease access to this almost exhaustive population research resource, through release as part of the “Health data hub” project. Finally, beyond these structured databases, the largest mine of untapped data worldwide remains the content of electronic health records (EHRs), encompassing a full range of data (clinical notes, laboratory results, imaging, genetic data, etc.) relating to patient care. Recent advances in information technology have made it easier for both hospitals and healthcare institutions to collect large amounts of healthcare data.

Biomedical scientists are now facing new challenges in the management and analysis of massive, heterogeneous datasets^56^. These challenges include the development of tools for exploration and visualization, analytical methods, integration into a comprehensive overview, and translation of the findings into public health impact. The visualization of information makes it possible for users to find profound patterns in clinical data, through visual recognition. Simple charts cannot represent the complexity of big data analyses and fail to support multifaceted tasks effectively^3,4^. There is, therefore, a need for sophisticated visualization tools dealing with many elements simultaneously and enabling users to perceive the patterns and insight generated by the algorithm^57^. Supplementary Table 1 shows the main data visualization tools used to present medical data. Many of the visual methods have been adopted directly from the field of data mining, but others, specific to the healthcare domain, have also been designed (Supplementary Table 2). For example, Happe and Drezen built the ePEPs toolbox, which displays relevant patterns extracted by eye from patient reimbursement data in the SNDS database, and supporting interactive exploration by researchers^58^. CARRE provides web-based components for interactive health data (fitness and biomarkers) visualization and risk analysis for the management of cardiorenal diseases^59^. The MITRE Corporation has also developed a web-based solution that provides an overview of an individual’s health through graphical representations of EHR data, highlighting abnormal values^60^. None of these visualization programs has yet managed to bridge the gap between of the large amounts of clinical data available and the discovery of clinical knowledge or paths for scientific research. By processing large heterogeneous sets of variables inherent to clinical records, MIIC provides physicians with a full picture of BC disease. It will be interesting to see how extending the present cohort of BC patients to larger BC cohorts treated with similar NAC therapy will allow us to refine the visual clinical network presented here, Fig. 1.

In addition to this use for visualization, the MIIC algorithm presents several other advantages for analyses, including its unsupervised nature, overcoming the need for training or human involvement. This feature makes it possible to obtain new knowledge through the automatic identification of patterns and dependences in the data, highlighting new interactions, and it may be of use for feature selection in machine learning models.

In conclusion, MIIC, an open-access, interactive, multitask tool, is designed to visualize datasets to help clinicians and researchers to understand the relationships between the variables within them. It opens up promising perspectives for guiding the generation of new hypotheses, helping clinicians identify actionable nodes and edges in clinical networks, and revealing new clues to relationships of interest for research purposes. Its widespread use in the field of health data could increase the accuracy of prediction for treatment responses and prognosis. This tool has the potential to improve the care pathway and, ultimately, the survival of patients.

## Methods

### Patients and treatment

We analyzed a cohort of 1197 patients with non-metastatic BC treated by NAC, with or without trastuzumab, followed by surgery, at either of the two Institut Curie sites (Paris and Saint Cloud) between 2002 and 2012 (NEOREP Cohort, CNIL declaration number 1547270. The methods were performed in accordance with relevant guidelines and regulations and approved by CNIL and the Breast Cancer Group of Institut Curie on September 11, 2020. Owing to the retrospective nature of this study, the ethics committee granted a waiver of informed consent for the included participants.). We included unilateral, non-recurrent, non-inflammatory, non-metastatic tumors, and excluded T4 tumors. This retrospective study was conducted in accordance with institutional and ethical rules regarding research on tissue specimens and patients. Information on family history, clinical characteristics (age; menopausal status; body mass index) and tumor characteristics (clinical tumor stage and grade; histology; clinical nodal status; ER, PR and *HER2* status; BC subtype; mitotic index; Ki67; lymphovascular invasion) was retrieved from electronic medical records. All the patients of the cohort received NAC, and additional treatments were decided in accordance with national guidelines.

### Tumor samples and pathological review

In accordance with the guidelines used in France (Group for Evaluation of Prognostic Factors using Immunohistochemistry in Breast Cancer^61^), cases were considered estrogen receptor (ER)-positive or progesterone receptor (PR)-positive if at least 10% of the tumor cells expressed estrogen and/or progesterone receptors (ER/PR). Endocrine therapy was prescribed when this threshold was exceeded. *HER2-*negative status was defined as a score of 0 or 1+ for the tissue section stained by immunohistochemistry (IHC). Tissue sections with scores of IHC 2+ or IHC 3+ were then analyzed by fluorescence in situ hybridization (FISH) to confirm *HER2* positivity. BC tumors were classified into subtypes (TNBC, *HER2*-positive, and luminal *HER2*-negative [referred to hereafter as “luminal”]). BC subtypes were defined as follows: luminal, ER^+^ or PR^+^/*HER2*^*−*^; TNBC, ER^−^/PR^−^/*HER2*^−^; *HER2*-positive BC, *HER2*^*+*^. Pretreatment core needle biopsy specimens and/or the corresponding post-NAC surgical specimens were reviewed independently by breast disease experts for research purposes, to assess residual cancer burden index, and the levels of tumor-infiltrating lymphocytes. The pathological reviews of these specimens are described in detail elsewhere^20,62,63^. Pathological complete response (pCR) was defined as the absence of residual invasive cancer cells in the breast and axillary lymph nodes (ypT0/is þ/ypN0).

### Survival endpoints

Relapse-free survival (RFS) was defined as the time from surgery to death, loco-regional recurrence or distant recurrence, whichever occurred first. Overall survival (OS) was defined as the time from surgery to death. The date of last known contact was retained for patients for whom none of these events were recorded. The cutoff date for survival analysis was March, 13th, 2019.

### Variables of interest

The care pathway of BC patients eligible for neoadjuvant chemotherapy can be summarized as follows: (*i*) pretreatment biopsy for BC diagnosis; (*ii*) administration of chemotherapy as the first-line treatment; (*iii*) removal of the tumor by surgery; (*iv*) histological analysis of the specimens obtained; (*v*) prescription of adjuvant treatments, if indicated (radiotherapy, hormonotherapy, chemotherapy); (*vi*) patients follow-up to monitor for relapse or death. We identified 94 clinically relevant variables from clinical, radiological, pathological and outcome data, which we grouped into 14 categories (hospital, history, comedication, comorbidities, clinical baseline, baseline histology, pre-NAC pathology, treatment response, surgery, treatment, changes during NAC, post-NAC pathology, delayed relapse/survival, metastasis). For composite variables derived from raw variables (e.g., BC subtype, constructed from a combination of ER status, PR status, *HER2* status), both derived and raw variables were represented on the network.

### MIIC algorithm

The functioning of the algorithm has been described in detail elsewhere^5,7^. Briefly, starting from a fully connected network, the MIIC algorithm first removes dispensable edges by iteratively subtracting the most significant information contributions from indirect paths between each pair of variables. The remaining edges, the underlying effect of which cannot be explained by indirect paths, are then oriented based on the causality signature in the data, corresponding to the simultaneous head-to-head orientations of so-called “v-structures”, X→Z←Y. In principle, propagation of v-structure orientations to downstream edges can also be implemented to fulfill underlying model class assumptions^64,65^ but are not applied on the NEOREP clinical network to ensure that MIIC algorithmic decisions are only based on information actually contained in the data.

Each edge corresponds to a “direct” association between two variables, that is, a statistical association that cannot be entirely explained by indirect effects involving other variables. Red and blue edges correspond to positive and negative (i.e., anti-correlated) associations, respectively. Four types of edge orientations are distinguished by the MIIC online server: (*i*) directed edges with a gray arrowhead represent inferred causal relationships; (*ii*) bidirected edges (drawn with dashed lines) reflect the presence of a latent common cause (*L*) unobserved in the available dataset, i.e.*, X*←(*L*)→*Y*; (*iii*) directed edges with a colored (red or blue) arrowhead are consistentent with either a causal or a latent common cause relationship; and (*iv*) undirected edges, whose orientation if it exists, cannot be inferred from non-perturbative data. The original algorithm was restricted to categorical variables^5^, but MIIC has recently been extended to include continuous variables, the values of which are partitioned into optimal bins, maximizing mutual information with another (continuous or categorical) variable of interest, while preventing the overfitting of datasets of finite size due to the use of too many bins^7^. In particular, each continuous variable may have different information-maximizing partitions depending on the associated variable of interest. For instance, MIIC finds three maximally informative bins for the residual cancer burden (RCB) score in association with patient survival status (Supplementary Fig. 1A), whereas eight RCB bins are required to estimate its mutual information with post-NAC cellularity correctly (Supplementary Fig. 1B).

### MIIC online server

The MIIC online server is freely accessible at https://miic.curie.fr and can be used with the Google Chrome, Mozilla Firefox, Edge, and Safari browsers. The user guide summarizing the main steps for running the MIIC algorithm is accessible at https://miic.curie.fr/user_guide.php, and an online video tutorial is available at: https://miic.curie.fr/tutorial.php. The workbench is available from https://miic.curie.fr/workbench.php. As input data, the user can upload a dataset formatted as a table with commas, semicolons, tabs, pipes or colons, as separators, without row names. Each variable can be either categorical or quantitative (discrete or continuous). Variables can be grouped into families, identified with different colors on the network. Missing values are allowed in the dataset and their possible statistical biases are taken into account by MIIC^7^. They should be indicated as “NA” in the dataset table. Once the dataset has been prepared, the user runs the algorithm, and an e-mail is sent when the job is completed.

### MIIC output

The MIIC online server generates a visualization of the global network of the dataset. An example based on the NEOREP dataset is displayed in Fig. 1, and is accessible as an interactive network at https://miic.curie.fr/job_results_showcase.php?id=NEOREP.

### Interactive exploration of the network

The distributions and neighborhoods of each node and edge of the inferred network can be explored through an interactive interface, through the mouse-over right- or left-click buttons on the browser page, as detailed in the online tutorials. Briefly, any variable can be highlighted by clicking on the network or through the “Search” toolbox (Supplementary Fig. 2A). The corresponding plots can be downloaded as.png or.svg images. Each node can be explored individually in terms of counts (categorical variables, Supplementary Fig. 2B, C) or distribution (continuous variables Supplementary Fig. 2D, E). Each edge can be explored by a right click and the choice of “plot join distribution” or “plot discretization”. The resulting plots are (i) proportion plots, with the edge representing the total association between two categorical variables (Supplementary Fig. 2F); (ii) distribution histograms (Supplementary Fig. 2G) or boxplots (Supplementary Fig. 2H), in which the edge represents the total association between a categorical and a continuous variable or (iii) scatter plots (Supplementary Fig. 2I), in which the edge represents the total association between two continuous variables. Additional options include inverting the *x* and *y* axes, the choice of frequency or absolute counts, or NA removal (proportion plots), and faceting or superimposing the variables (distribution histograms). All the figures presented here were generated with the MIIC online interactive visualization tool.

### Reporting summary

Further information on research design is available in the Nature Research Reporting Summary linked to this article.

## Supplementary information

Supplementary Material

Reporting Summary

## Data Availability

All images and the associated network are publicly available at: https://miic.curie.fr/job_results_showcase.php?id=NEOREP. Data corresponding to the NEOREP cohort study will be available upon reasonable request.

## Code availability

The code corresponding to the MIIC algorithm can be found at the following address: https://github.com/miicTeam/miic_R_package.
