# Supplementary material for: Interactive exploration of a global clinical network from a large breast cancer cohort
Source: NPJ Digit Med. 2022 Aug 10;5:113. doi: 10.1038/s41746-022-00647-0 (PMC9365762; doi:10.1038/s41746-022-00647-0)
Supplement: Supplementary file 1 — Supplementary Material [file 41746_2022_647_MOESM1_ESM.docx]

**Supplementary information file**

**Interactive exploration of a global clinical network from a large breast cancer cohort**

Nadir Sella, Anne-Sophie Hamy, Vincent Cabeli, Lauren Darrigues, Marick Laé, Fabien Reyal, Hervé Isambert

**Supplementary Figures**

**
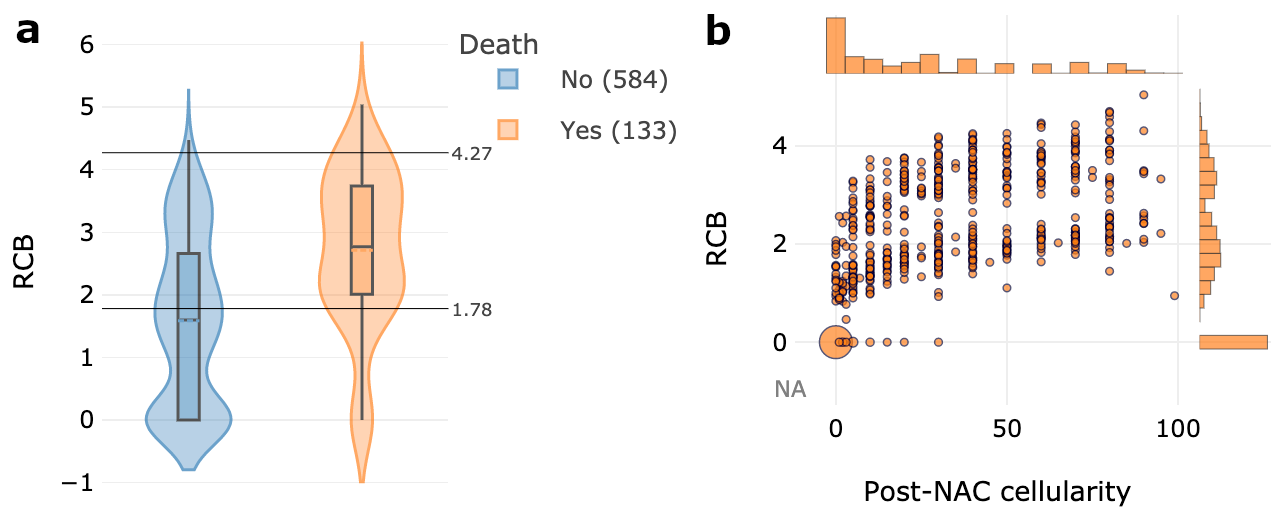
**

**Supplementary Figure 1. The extended MIIC algorithm maximizes the mutual information between continuous or mixed-type variables.**

a) MIIC finds three bins for the variable RCB when optimizing the mutual information with variable death. The boxes represent the IQR, the horizontal lines correspond to the median values and lines ends mark the upper and lower fence. The number of cases considered for the analysis are reported in the legend. b) MIIC finds eight bins for the variable RCB when optimizing the mutual information with the variable post-NAC cellularity.


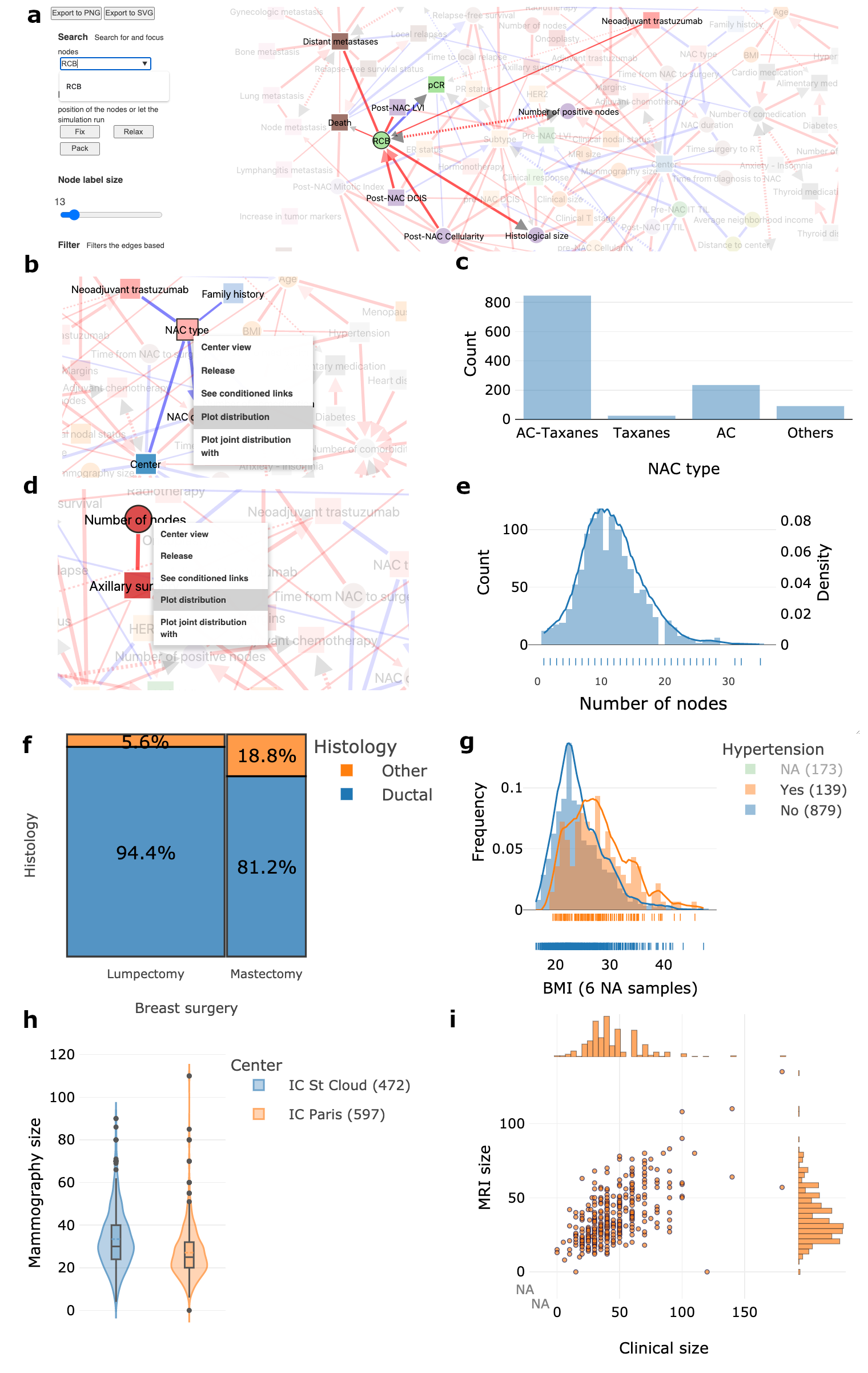


**Supplementary Figure 2. Interactive exploration of the network.**

a) Nodes within the network are highlighted when mentioned and selected in the search toolbox. b-c) Counts by level of a categorical variable (squared nodes) are displayed by right clicking on the “node → plot distribution” option. d-e) The distribution of each continuous variable (round nodes) is displayed by right clicking on the “node → plot distribution” option. f) Proportion plot or treemaps plot displaying the relationships between two categorical variables, available by right clicking on the “edge→ plot joint distribution” option. g) Distribution plot displaying the relationships between one categorical variable and one continuous variable, available by right clicking on the “edge→ plot joint distribution” option. h) Boxplot displaying the relationships between one categorical variable and one continuous variable, available by right clicking on the “edge→ plot discretization” option. The boxes represent the IQR, the horizontal lines correspond to the median values and lines ends mark the upper and lower fence. The number of cases considered for the analysis are reported in the legend. i) Scatterplot displaying the relationships between two continuous variables, available by right clicking on the “edge→ plot joint distribution” option.


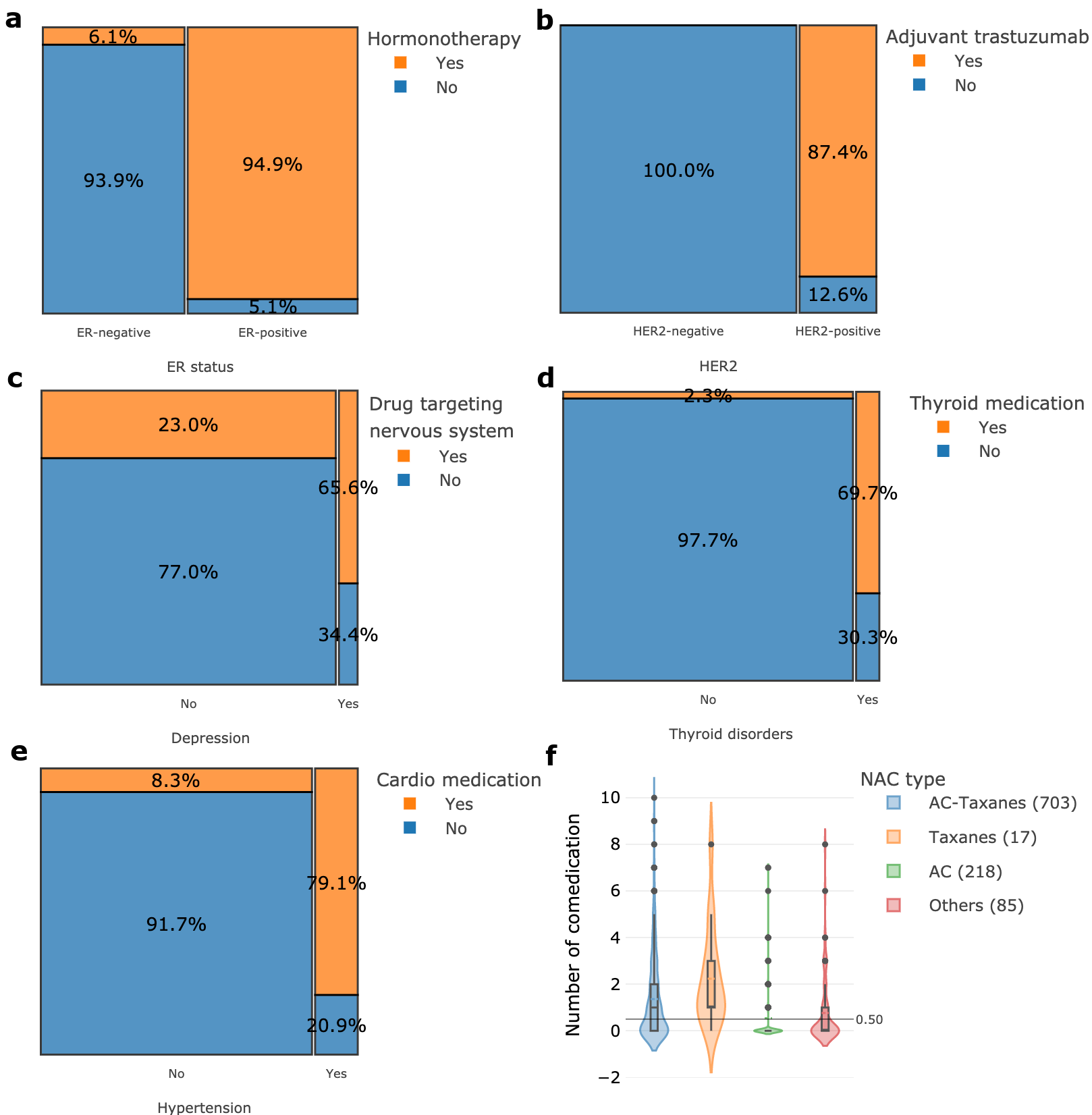


**Supplementary Figure 3.** **MIIC identifies relationships between a disease and the corresponding treatment.**

a) Proportion plot for adjuvant hormonotherapy and tumor estrogen receptor (ER) status .b) Proportion plot for adjuvant trastuzumab and tumor HER2 status. c) Proportion plot for depression and psychoactive medication. d) Proportion plot for thyroid disorders and thyroid medication. e) Proportion plot for arterial hypertension and cardiac medication. f) Distribution of neoadjuvant chemotherapy (NAC) type by number of drugs taken by the same patient (comedication). The boxes represent the IQR, the horizontal lines correspond to the median values and lines ends mark the upper and lower fence. The number of cases considered for the analysis are reported in the legend.

**
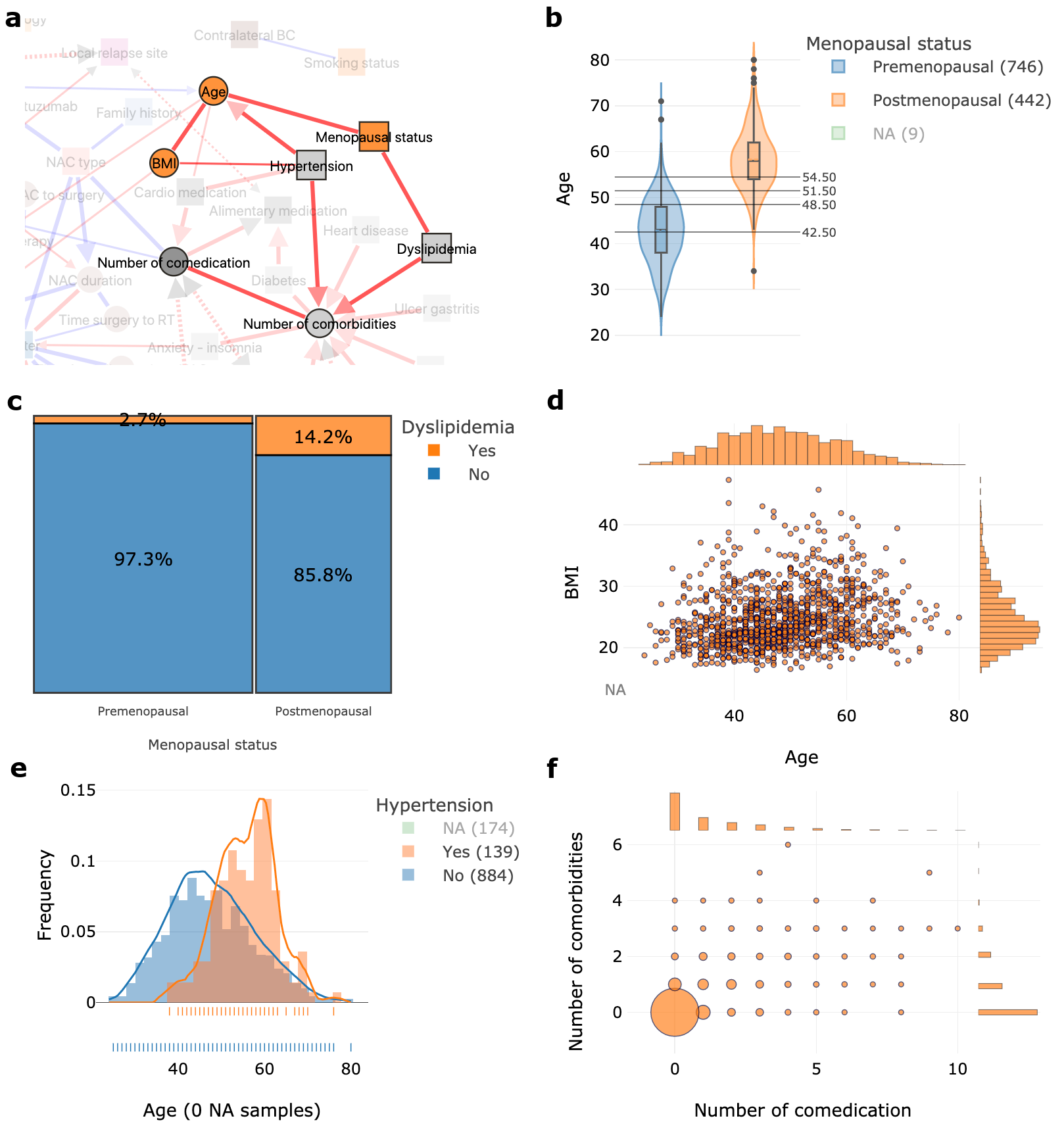
**

**Supplementary Figure 4.** **MIIC identifies factors known to be epidemiologically associated.**

a) Menopausal status is connected to the patient’s age and dyslipidemia; hypertension is linked to age and body mass index (BMI); and the number of comorbidities is linked to the number of drugs taken (comedication). b) Boxplot comparing the age distribution of premenopausal (*N*=746) and postmenopausal patients (*N*=442). The boxes represent the IQR, the horizontal lines correspond to the median values and lines ends mark the upper and lower fence. The number of cases considered for the analysis are reported in the legend. c) Proportion plot for menopausal status and dyslipidemia. d) Scatter plot for BMI and patient age: the black lines correspond to the median values; density proportions are shown at the top and side of the graph. e) Distribution plot showing patient age as a function of the presence or absence of hypertension. f) Scatter plot between the number of patient comorbidities and the number of drugs taken by the patient (comedication).


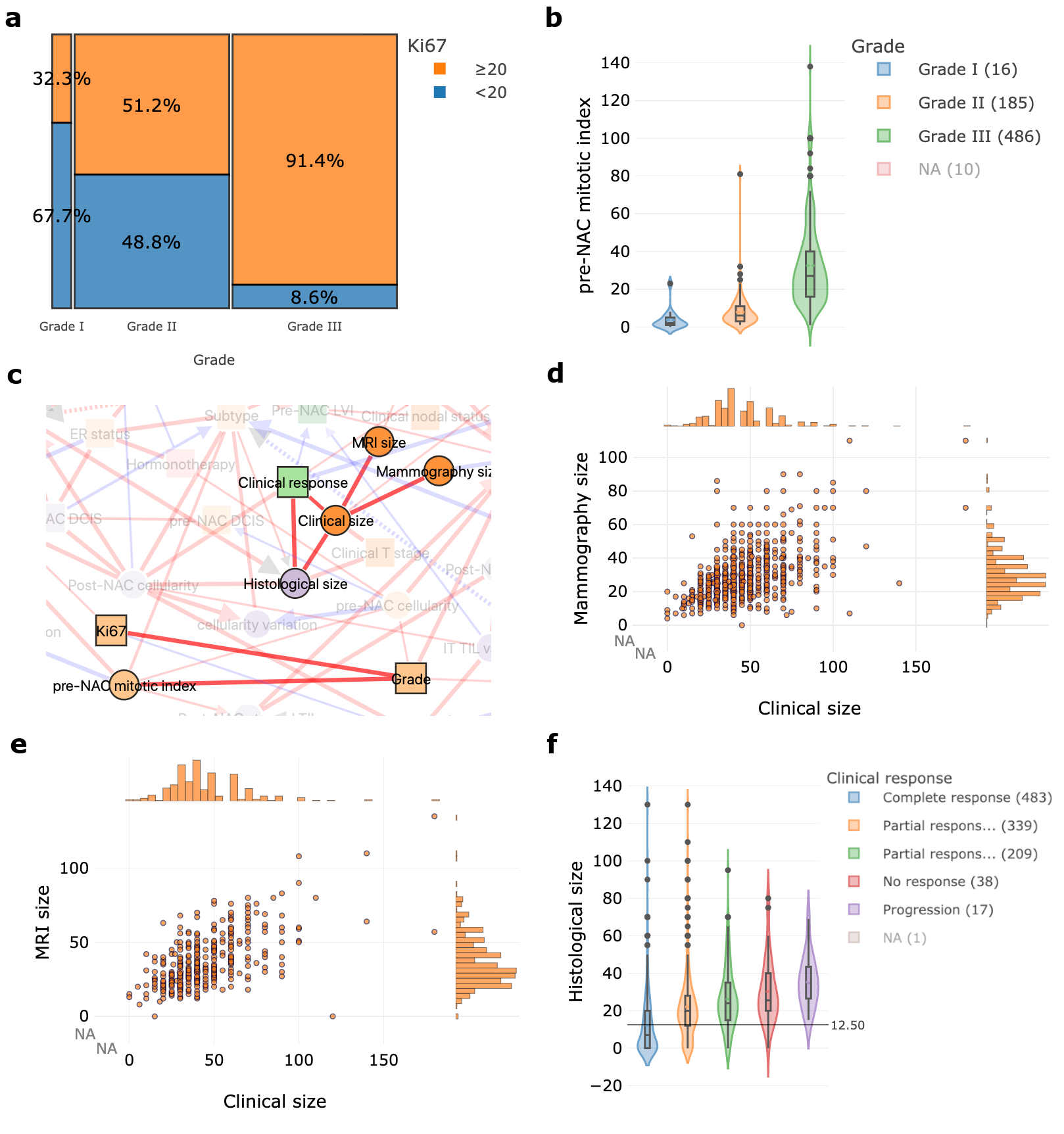


**Supplementary Figure 5.** **MIIC identifies intra- and inter-modality associations.**

a) Proportion plot for tumor grade (I, II and III) and percentage of cells expressing Ki67 (< or > to 20%) for each patient. b) Boxplots displaying pre-NAC mitotic index values by tumor SBR grade. c) Network interactions around node clinical size and tumor grade. Clinical size is linked to sizes on MRI, mammography and histological analysis, but also to clinical response. Tumor grade is linked to Ki67 and pre-NAC mitotic index. d) Scatter plot showing the correspondence between clinical size and size on mammography for the tumor of each patient. e) Scatter plot showing the correspondence between clinical size and size on MRI for the tumor of each patient. f) Boxplot showing the correlation between tumor size on histological analysis and clinical response (complete response to progression). The boxes represent the IQR, the horizontal lines correspond to the median values and lines ends mark the upper and lower fence. The number of cases considered for the analysis are reported in the legend.


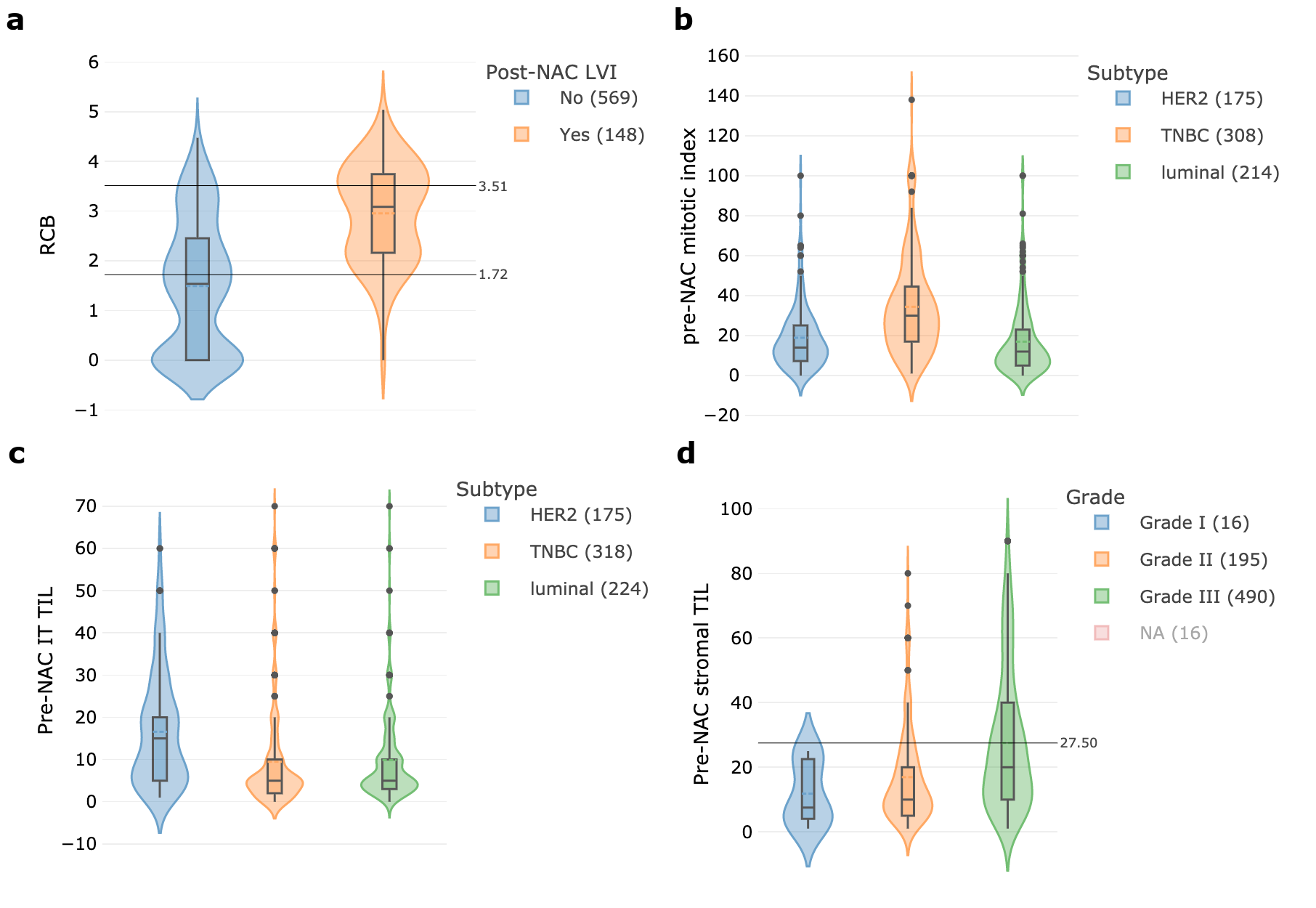


**Supplementary Figure 6.** **MIIC provides insight into tumor biology and response to treatment.**

a) Boxplot comparing the distribution of RCB values according to the presence of post-NAC lymphovascular invasion: the median value for RCB is 3.51 in the presence of LVI and 1.72 in its absence. b) Boxplot comparing the distribution of pre-NAC mitotic index (MI) between tumor subtypes. c) Boxplot comparing pre-NAC stromal TIL distribution between tumor subtypes. d) Boxplot comparing pre-NAC stromal TIL distribution between tumor grades. The boxes represent the IQR, the horizontal lines correspond to the median values and lines ends mark the upper and lower fence. The number of cases considered for the analysis are reported in the legend.


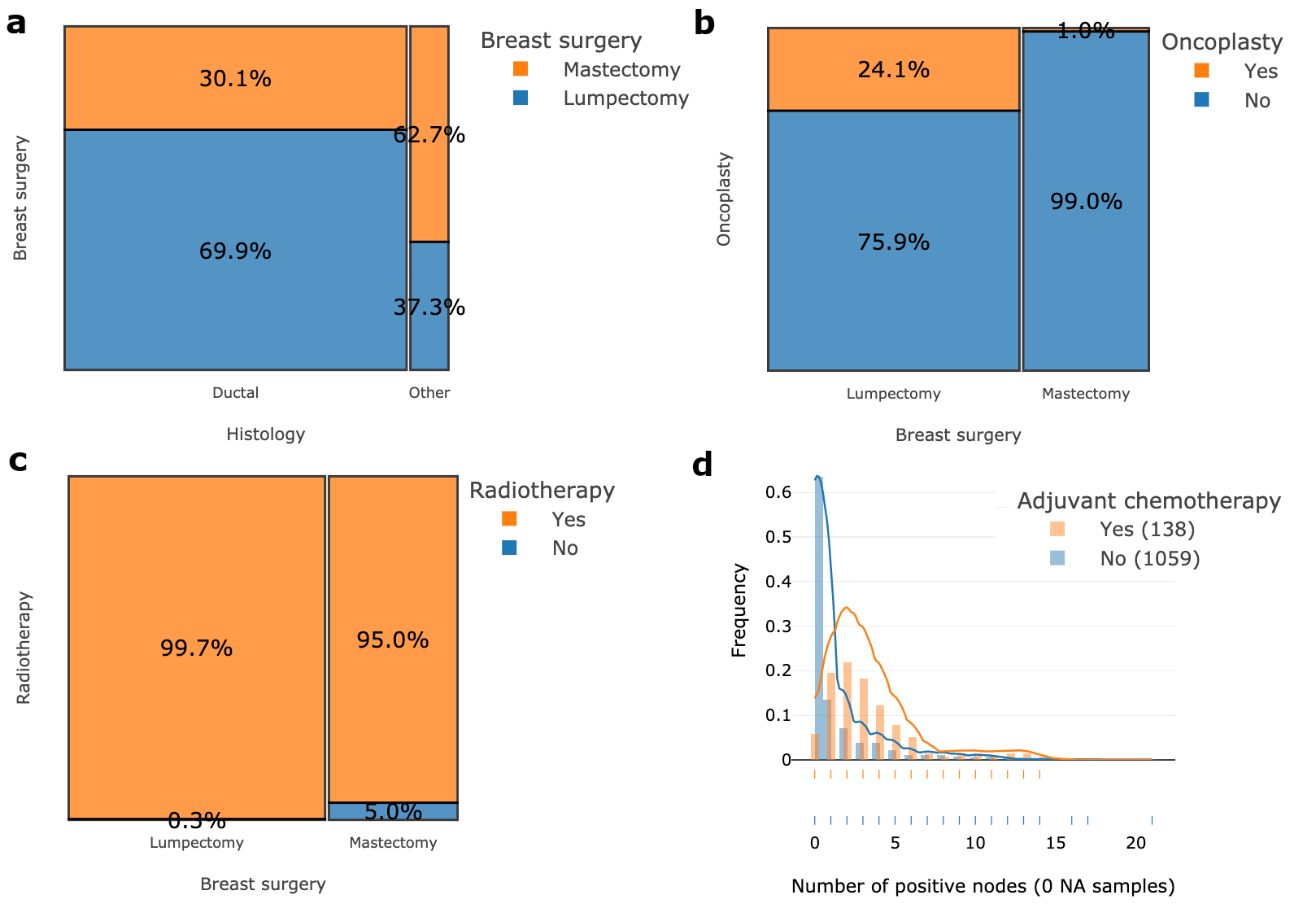


**Supplementary Figure 7. MIIC reflects clinical practice.**

a) Proportions of lumpectomies and mastectomies performed, according to the histological type of breast cancer, ductal or other (lobular). b) Proportion of conservative surgery interventions as a function of oncoplastic surgery c). Proportion of patients receiving adjuvant radiotherapy according to the type of breast surgery performed: lumpectomy or mastectomy. d) Boxplot comparing numbers of positive axillary lymph nodes in patients with and without adjuvant chemotherapy.

**Supplementary Tables**

**Supplementary Table 1. Visualization and graphical tools often used to represent medical data analyzes.**

| **Datavisualization tools** | **Description** | **Applications** |
| --- | --- | --- |
| **Parallel coordinates** | - it displays M equally spaced vertical axes with individual ranges corresponding to one clinical variable. Each of the N patient are illustrated as a line that passes through each of the axes.  - allows the users to interactively define a range within a single or multiple variables (axes), explore correlations between variables for the selected patients, look for outliers. | *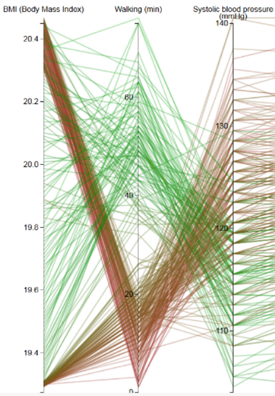*  *Example in where negative correlations can be found between walking minutes, PAs as well as BMI (Luo et al., 2016)*^1^ |
| **Chord visualization plot** | - circular plot consisting of boundaries, where each boundary is a variable. Connections are drawn between boundaries and the thickness of each connection represents the strength of the correlation. | 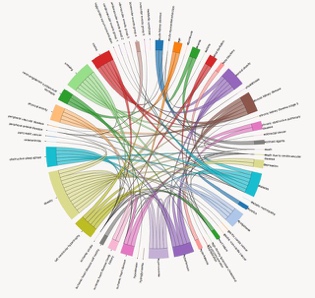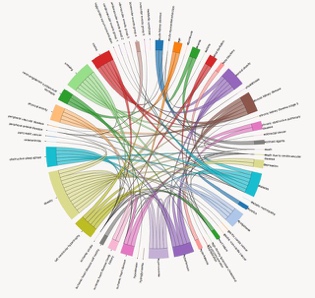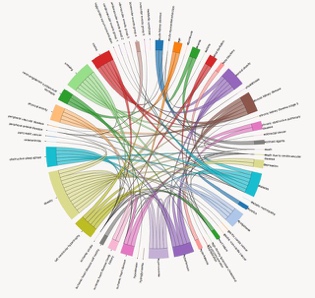  *`*  *-ex : Risk elements are placed on the circle and the chords represent the risk associations. Relationships of all risk elements can be clearly presented with CARRE* ^2^ |
| **The star glyphe** | - constructed by creating M axes for each variable following a radial configuration : the value for each variable is normalized between 0.0 and 1.0 and drawn along the axe. | 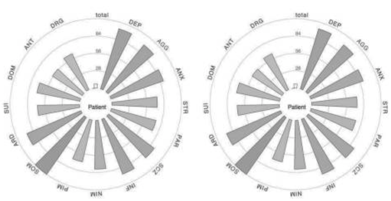 **Patient 1**  **Patient 2**  *Comparison of multiple clinical elements between two different patients, here we can see that the two subjects follow a similar distribution, thus resulting in a similar shape* |
| **Hierarchical trees and treemaps** | - displays hierarchical data as a set of nested rectangles. Each branch  of the tree is given a rectangle, then tiled with smaller rectangles representing sub-branches. A leaf node's rectangle has an area proportional to a specified dimension of the data  - the leaf nodes are colored to show a separate dimension of the data. | 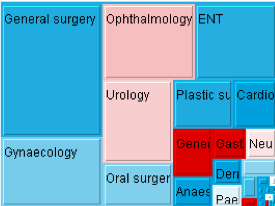  *Treemap showing changes in waiting times for patients of English PCTs: size shows the capacity and color shows the waiting time* |
| **Bayesian network** | - model of a multivariate probability distribution network structure over a set of random variables;  - each node in the graph represents a random variable, while the edges between the nodes represent probabilistic dependencies among the corresponding random variables. | 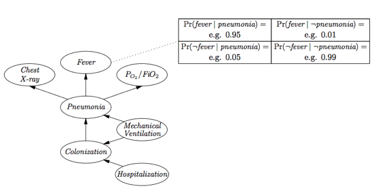  *Network structure which encodes dependence and independence information among the random variables, and a set of local probability distribution tables.* |

**Supplementary Table 2. Visualization systems for health data exploration.**

| **Datavisualization Tools** | **Data** | **Abilities** | **Limitations** |
| --- | --- | --- | --- |
| **ePEPS toolbox**  ^3^ | French nationwide health record database (SNDS) | -subsets group of patients according to events in their healthcare pathways  -produces basic statistical indicators of the groups through visual outputs  -creates interactive visual representation of the individual patient trajectories | Constrained to SNDS database only  No statistical analyzes |
| **CARRE Visual Analytics**  ^2^ | Fitness data from sensors/apps, biomarker data from PHR and risk factor data from medical literatures. | - health monitoring with visual analytics components of timeline and parallel coordinates  - risk analysis using node-link, chords or sanker diagrams  - multiple variable time-dependent data (fitness, biomarkers) visualized in a linear healthline helps to study | Limited to cardiorenal disease comorbidities Based on risk models extracted from medical publications. |
| **MyHealthAvatar**  ^2^ | Fitness and lifestyle data automatically collected or manually input | -health self-monitoring, including fitness and spatial-temporal lifestyle data represented with timelines, clock views, maps and activity graphs  - health data collection, storage and access for patients, doctors and nonmedical users. | Lifestyle analysis oriented  Highly dependent on relationship visualization and analysis  health self-monitoring |
| **hGraph**  MITRE Corporation  ^4^ | Laboratory tests, physical activity, nutrition, sleep monitors found in PHI, EHR and PHR | - a single source representation of a person’s overall health state : values are distributed in a circular space  - provides a quick overview of the general situation of all the values and how they deviate from the recommendations. | Health self-monitoring  Based on pre-recorded recommendations |
| **Tableau**  ^5^ | Preprocessed excel data | - connects multiple datasets to generate a lot of views in one workbook and share the data filters and markers  - automatically construct graphs, charts, maps, and timelines as table visualizations  - graphical presentation of quantitative and categorical information. | A strict visualization tool  Poor in business intelligence: need implementation for analysis  High cost |
| **Gnaeus**  ^5^ | EHR data | - utilizes clinical guidelines for knowledge-assisted visualization of EHR cohorts | Does not assist in finding the cohort |
| **HARVEST**  ^6^ | Clinical and laboratory data | - visualizes individual patients’ longitudinal medical history  -provides interactive, problem-oriented patient record summarization system.  - has natural language parser of the patient notes and aggregates and presents information from multiple care settings. | Clinical questions hiding in cohort or relying on clinical note will be missing.  Constrained to one medical center |
| **EventFlow**  ^7^ | EHR, claims data, codes extracted from video observations of medical procedures. | - visualizes and review the data from individual records and their event sequence  - searches for temporal patterns of interest, using graphical interface;  - summarizes all the event sequences, their timing and prevalence  - selects cohorts of interest for further studies. | Clinical researchers still need a self-service tool for preparing the data before they simplify and find patterns in it  High cost |

**Supplementary References**

**1**. Luo J, Wu M, Gopukumar D, et al: Big Data Application in Biomedical Research and Health Care: A Literature Review. Biomed Inform Insights 8:1–10, 2016

**2**. Zhao Y, Parvinzamir F, Wei H, et al: Visual Analytics for Health Monitoring and Risk Management in CARRE. E-Learning and Games; 10th International Conference, Edutainment 2016, Hangzhou, China, April 14-16, 2016, Revised Selected Papers 9654:380–391, 2016

**3**. Happe A, Drezen E: A visual approach of care pathways from the French nationwide SNDS database - from population to individual records: the ePEPS toolbox [Internet], 2018[cited 2019 Aug 18] Available from: https://hal-univ-rennes1.archives-ouvertes.fr/hal-01697626

**4**. Ledesma A, Al-Musawi M, Nieminen H: Health figures: an open source JavaScript library for health data visualization [Internet]. BMC Med Inform Decis Mak 16, 2016[cited 2019 Aug 14] Available from: https://www.ncbi.nlm.nih.gov/pmc/articles/PMC4802654/

**5**. Ko I, Chang H: Interactive Visualization of Healthcare Data Using Tableau. Healthc Inform Res 23:349–354, 2017

**6**. Federico P, Unger J, Amor-Amorós A, et al: Gnaeus: utilizing clinical guidelines for knowledge-assisted visualisation of EHR cohorts5

**7**. Monroe M, Lan R, Lee H, et al: Temporal event sequence simplification. IEEE Trans Vis Comput Graph 19:2227–2236, 2013
